# Shape complexity in cluster analysis

**DOI:** 10.1371/journal.pone.0286312

**Published:** 2023-05-26

**Authors:** Eduardo J. Aguilar, Valmir C. Barbosa

**Affiliations:** 1 Instituto de Ciência e Tecnologia, Universidade Federal de Alfenas, Poços de Caldas, MG, Brazil; 2 Programa de Engenharia de Sistemas e Computação, COPPE, Universidade Federal do Rio de Janeiro, Rio de Janeiro, RJ, Brazil; Naval Postgraduate School, UNITED STATES

## Abstract

In cluster analysis, a common first step is to scale the data aiming to better partition them into clusters. Even though many different techniques have throughout many years been introduced to this end, it is probably fair to say that the workhorse in this preprocessing phase has been to divide the data by the standard deviation along each dimension. Like division by the standard deviation, the great majority of scaling techniques can be said to have roots in some sort of statistical take on the data. Here we explore the use of multidimensional shapes of data, aiming to obtain scaling factors for use prior to clustering by some method, like k-means, that makes explicit use of distances between samples. We borrow from the field of cosmology and related areas the recently introduced notion of shape complexity, which in the variant we use is a relatively simple, data-dependent nonlinear function that we show can be used to help with the determination of appropriate scaling factors. Focusing on what might be called “midrange” distances, we formulate a constrained nonlinear programming problem and use it to produce candidate scaling-factor sets that can be sifted on the basis of further considerations of the data, say via expert knowledge. We give results on some iconic data sets, highlighting the strengths and potential weaknesses of the new approach. These results are generally positive across all the data sets used.

## Introduction

The common wisdom regarding the processing of data prior to cluster analysis, particularly when a distance-based clustering method like k-means or some forms of hierarchical clustering are used, is that data should be scaled to improve results. Even though researchers have been prolific in creating domain-specific forms of scaling (cf., e.g., [1]), already the earliest studies systematically approaching the subject viewed division by the standard deviation or by the range in each dimension as the natural candidates they still are to this day [2, 3]. This is not to say that alternative divisors were not considered: they were [4], but the situation seems to have remained largely unchanged until very recently, with the introduction of the so-called pooled standard deviation [5], which continues to support division by the standard deviation unless this would make the dimension in question lose information crucial to partitioning the data into clusters. Should this be the case, a weighted averaged of the standard deviations localized around statistically significant modes in that dimension is used instead. This average is the pooled standard deviation of the data in that dimension, henceforth denoted by $\sigma_{k}^{\text{pool}}$ for dimension *k*. Notably, the essential motivation for the creation of $\sigma_{k}^{\text{pool}}$ seems well aligned with concerns that have been voiced since the late 1960’s (cf. [3] for comments on this).

In a similar vein, it has for several decades been clear that some form of optimization problem must exist whose solution yields scaling factors that make some sort of sense for the various dimensions. And indeed this has been pursued, though to the best of our knowledge not for the last three decades, at least. Noteworthy representatives of these attempts include optimizing for a linear transformation of the data [6]; maximizing the square of a correlation between two sets of distances between samples [7]; a least-squares method for determining scaling factors that make such distances approach those in the dendrogram resulting from hierarchical clustering [8]; and determining scaling factors by considering the modal structure of the data in each dimension in a way that, to a certain degree, prefigures the above definition of the pooled standard deviation [9]. Each of these approaches seems to have either disappointed its own creator [6], or remained tailored to the generally uninteresting cases of nonoverlapping clusters [7], or been tested only superficially [8], or simply remained untested [9].

Here we introduce the use of a shape-complexity function to guide the determination of scaling factors. By this denomination we are not referring to one of the many forms of complexity used to characterize the computer representations of three-dimensional shapes [10]. Instead, we refer to a generalization to multiple dimensions of the homonymous three-dimensional concept introduced recently in cosmology and related disciplines [11, 12]. If we imagine (up to three dimensions) that the disposition of data samples in space gives the data set an inherent shape, then clearly being able to shrink or stretch each dimension independently of all others is an important source of shape variation, one that we explore for the purpose of cluster analysis. One particular facet of shape complexity that we find especially relevant to this end is that it allows what should be intercluster distances to be considered side-by-side with distances that should be intracluster. While normally cluster analysts know which distance is which type only in a very limited manner, shape complexity provides a handle that can help in posing a nonlinear programming problem for the automatic determination of scaling factors (or rather, candidate scaling factors to undergo further scrutiny based on what analysts do know of the domain in question).

We next introduce the form of shape complexity we use, giving its definition and properties of interest. We also discuss why it relates closely to the role of scaling factors in cluster analysis and how determining such factors from it can be formulated. We then proceed with a description of our experimental setup. This includes the data sets on which we experiment, the tools and algorithms we use, and how we evaluate a data set’s partition into clusters. Importantly, we perform clustering solely via the k-means method, owing mainly to its great potential to perform well when clusters overlap [13], and also to its long history, during which many implementations and variants have appeared [14].

## Shape complexity

We consider an *n*_orig_ × *d* data matrix *X* with *n*_orig_, *d* > 1, where *n*_orig_ is the number of *d*-dimensional real samples. For 1 ≤ *k* ≤ *d*, we use $\sigma_{k}^{2}$ to denote the samples’ variance on dimension *k*, and *α*_*k*_ > 0 to denote the scaling factor to be used on the samples along this dimension in order to facilitate clustering. Factor *α*_*k*_ is assumed to be applied to the various *X*_*ik*_’s along with their division by *σ*_*k*_. That is, each original *X*_*ik*_ is to undergo scaling by the factor *α*_*k*_/*σ*_*k*_. This makes it easier to assess the effect of factor *α*_*k*_ relative to the more common 1/*σ*_*k*_ and also enables some key developments later on.

For reasons to be discussed shortly, here we propose that the appropriate *α*_*k*_’s be determined with the guidance of the so-called shape complexity of the *n*_orig_ points in *d*-dimensional real space that define the samples. This notion is borrowed from the physics of multiple bodies interacting gravitationally with one another. For *d* = 3 and the points having masses associated with them, shape complexity has been shown to help account for structure as it arises in the form of clusters during the system’s evolution [11, 12].

The version of shape complexity we use, denoted by SC, is given by

$$\begin{array}{c} \text{SC}={\left(\sum_{i<j}r_{ij}^{2}\right)}^{1/2}\sum_{i<j}r_{ij}^{-1}, \end{array}$$ (1)

where each *i* and *j* are distinct samples and *r*_*ij*_ is the Euclidean distance between them. That is, the number of samples SC takes into account is *n* such that 1 < *n* ≤ *n*_orig_ (duplicates may thus exist only if *n*_orig_ > 2) and

$$\begin{array}{c} r_{ij}^{2}=\sum_{k}\alpha_{k}^{2}\rho_{ijk}^{2}, \end{array}$$ (2)

with

$$\begin{array}{c} \rho_{ijk}=\frac{X_{ik}-X_{jk}}{\sigma_{k}}. \end{array}$$ (3)

SC is therefore a function of the *α*_*k*_’s, but we refrain from denoting this explicitly for the sake of notational clarity. Importantly, the use of *i* < *j* in the summations of Eq (1) indicates that they occur on the set of all $\left(\begin{array}{c} n\\ 2 \end{array}\right)$ unordered pairs of distinct samples. Likewise, the summation on *k* in Eq (2) indicates that it occurs over all *d* dimensions.

### Radial invariance

One of the key properties for which SC is appreciated in its fields of origin is scale invariance, which in our terms is to be understood as follows. If *α*_*k*_ has the same value for every *k*, then clearly SC remains unchanged however this common value is varied. But if SC is to be used to improve the results of clustering algorithms on the data, setting every *α*_*k*_ to the same value is in general not an option. Scale invariance, nevertheless, is a special case of the much more useful radial invariance we discuss next. The radial invariance of SC can be seen in more than one way, but here we choose the perspective of certain directional derivatives of SC. This requires us to already consider the gradient of SC, which will be instrumental later on.

For *f* a differentiable function of the *α*_*k*_’s, we let $f_{k}^{\prime }$ denote ∂*f*/∂*α*_*k*_, the *k*th component of the gradient of *f*, and moreover write $g={\left(\sum_{i<j}r_{ij}^{2}\right)}^{1/2}$ and $h=\sum_{i<j}r_{ij}^{-1}$ so that SC = *gh*. We get

$$\begin{array}{c} {\text{SC}}_{k}^{\prime }=g_{k}^{\prime }h+gh_{k}^{\prime }, \end{array}$$ (4)

where

$$\begin{array}{c} g_{k}^{\prime }=\alpha_{k}g^{-1}\sum_{i<j}\rho_{ijk}^{2} \end{array}$$ (5)

and

$$\begin{array}{c} h_{k}^{\prime }=-\alpha_{k}\sum_{i<j}r_{ij}^{-3}\rho_{ijk}^{2}. \end{array}$$ (6)

Radial invariance comes from realizing that the directional derivative of SC is zero along any straight line extending out from the origin into the positive *d*-dimensional real orthant, that is,

$$\begin{array}{c} \sum_{k}\alpha_{k}{\text{SC}}_{k}^{\prime }=0 \end{array}$$ (7)

for any valuation of the *α*_*k*_’s. To put it differently, SC has the same value at any two assignments of values to *α*_1_, …, *α*_*d*_, say $v_{1}^{\left(1\right)},\ldots ,v_{d}^{\left(1\right)}$ and $v_{1}^{\left(2\right)},\ldots ,v_{d}^{\left(2\right)}$, such that $v_{k}^{\left(2\right)}=tv_{k}^{\left(1\right)}$ for every *k* and some *t* > 0.

To see how Eq (7) comes about, simply write

$$\begin{array}{c} g\sum_{k}\alpha_{k}{\text{SC}}_{k}^{\prime }=h\sum_{k}\alpha_{k}^{2}\sum_{i<j}\rho_{ijk}^{2}-g^{2}\sum_{k}\alpha_{k}^{2}\sum_{i<j}r_{ij}^{-3}\rho_{ijk}^{2} \end{array}$$ (8)

$$\begin{array}{cc} & =h\sum_{i<j}\sum_{k}\alpha_{k}^{2}\rho_{ijk}^{2}-g^{2}\sum_{i<j}r_{ij}^{-3}\sum_{k}\alpha_{k}^{2}\rho_{ijk}^{2} \end{array}$$ (9)

$$\begin{array}{cc} & =h\sum_{i<j}r_{ij}^{2}-g^{2}\sum_{i<j}r_{ij}^{-3}r_{ij}^{2} \end{array}$$ (10)

$$\begin{array}{cc} & =hg^{2}-g^{2}h. \end{array}$$ (11)

The role of Eq (2) in this development highlights a condition equivalent to radial invariance: that the value of any *r*_*ij*_ becomes scaled by *t* when moving from $v_{1}^{\left(1\right)},\ldots ,v_{d}^{\left(1\right)}$ to $v_{1}^{\left(2\right)},\ldots ,v_{d}^{\left(2\right)}$.

### Shape complexity and clustering

Increasing any *r*_*ij*_ always increases *g* while decreasing *h*. Notably, the most significant increases in *g* come from increasing the largest *r*_*ij*_’s (since ∂*g*/∂*r*_*ij*_ = *g*^−1^*r*_*ij*_), while the most significant decreases in *h* come from increasing the smallest *r*_*ij*_’s (since $\partial h/\partial r_{ij}=-r_{ij}^{-2}$). Because increases in the *r*_*ij*_’s are mediated by increases in the *α*_*k*_’s, the effect of increasing any specific *α*_*k*_ on the *r*_*ij*_’s of specific relative magnitudes is best understood by considering how the ratios $g_{k}^{\prime }/g$ and $-h_{k}^{\prime }/h$ relate to each other. Two cases must be considered, as follows.

C1 If $g_{k}^{\prime }/g>-h_{k}^{\prime }/h$ (i.e., increasing *α*_*k*_ causes more of a relative increase in *g* than a relative decrease in *h*), then larger *r*_*ij*_’s are being increased more than smaller *r*_*ij*_’s.
C2 If $g_{k}^{\prime }/g<-h_{k}^{\prime }/h$ (i.e., increasing *α*_*k*_ causes more of a relative decrease in *h* than a relative increase in *g*), then smaller *r*_*ij*_’s are being increased more than larger *r*_*ij*_’s.

In the context of data clustering, assume for a moment that larger *r*_*ij*_’s are generally intercluster distances while smaller *r*_*ij*_’s are generally intracluster distances. Cases C1 and C2 above are then in strong opposition to each other, as clearly case C1 could be good for clustering and case C2 bad for clustering. We might then expect to be well-off if we targeted case C1 for every *k*, but surely an assignment of values to *α*_1_, …, *α*_*d*_ might satisfy case C1 for a specific *k* while satisfying case C2 for another. In this case it would seem better to pursue the intermediate goal of getting as close as possible to achieving $g_{k}^{\prime }/g=-h_{k}^{\prime }/h$ for every *k*.

Real-world data, however, rarely comply with the dichotomy we momentarily assumed above. Instead, quite often larger distances are intracluster, and likewise smaller distances are intercluster. In any case, the centerpiece of the strategy we adopt henceforth is the same that would be appropriate had the dichotomy always held true, that is, seeking the equilibrium represented by $g_{k}^{\prime }/g=-h_{k}^{\prime }/h$ for every *k*. On top of this, we essentially look for several scaling-factor schemes approaching such conditions as closely as possible and select the one (or more than one) that upon closer inspection of the data leads to a reasonable partition into clusters.

### The optimization problem

A consequence of our discussion of the radial-invariance property of SC is that all assignments of values to *α*_1_, …, *α*_*d*_ on any straight line emanating from the origin into the positive *d*-dimensional real orthant are equivalent at providing scaling factors for distance-based clustering. That is, choosing any such assignment will lead any distance-based clustering algorithm to yield the same result. This follows from the fact that the *r*_*ij*_’s for a given assignment on that line are scaled versions, by the same factor on all dimensions, of *r*_*ij*_’s for any of the other assignments.

In what follows, all but one of such equivalent assignments are ignored. The one that is taken into account is that for which

$$\begin{array}{c} \sum_{k}\alpha_{k}^{2}=d. \end{array}$$ (12)

That is, valid assignments of values to the *α*_*k*_’s must be on the *d*-dimensional sphere of radius $\sqrt{d}$ centered at the origin. This choice of radius allows for *α*_*k*_ = 1 for every *k* to be a valid assignment. This, we recall, is the assignment that scales the data along dimension *k* by the factor 1/*σ*_*k*_.

Seeking to approximate $g_{k}^{\prime }/g=-h_{k}^{\prime }/h$ for every *k* given this equality constraint boils down to the problem of finding a local minimum or maximum of SC given the constraint. Because SC is inextricably based on the data to be clustered, it seems to have no characteristic that can be directly exploited to this end. We follow an indirect route and begin by considering the first-order necessary condition for local optimality in this case [15], which requires not only the equality constraint in Eq (12) to be satisfied but also the gradient of the corresponding Lagrangian with respect to the *α*_*k*_’s to equal zero. The Lagrangian in this case is

$$\begin{array}{c} L=\text{SC}+\lambda (\sum_{k}\alpha_{k}^{2}-d), \end{array}$$ (13)

where λ is the Lagrange multiplier corresponding to the single equality constraint. Its gradient’s *k*th component is $L_{k}^{\prime }=g_{k}^{\prime }h+gh_{k}^{\prime }+2\lambda \alpha_{k}$. Writing this in more detail yields

$$\begin{array}{c} L_{k}^{\prime }=\alpha_{k}(g^{-1}h\sum_{i<j}\rho_{ijk}^{2}-g\sum_{i<j}r_{ij}^{-3}\rho_{ijk}^{2}+2\lambda ), \end{array}$$ (14)

from which it follows that, in order to achieve $L_{k}^{\prime }=0$ for every *k*, we must have

$$\begin{array}{c} \sum_{i<j}r_{ij}^{-3}\frac{\rho_{ijk}^{2}}{\sum_{i<j}\rho_{ijk}^{2}}=\frac{g^{-1}h+2\lambda {\left(\sum_{i<j}\rho_{ijk}^{2}\right)}^{-1}}{g} \end{array}$$ (15)

for each of them.

Even though it would seem that the right-hand side of Eq (15) may have a different value for each *k*, letting *N* = *n*_orig_(*n*_orig_ − 1) we note that $\sigma_{k}^{2}$ can be written as

$$\begin{array}{c} \sigma_{k}^{2}=N^{-1}\sum_{i<j}{\left(X_{ik}-X_{jk}\right)}^{2}, \end{array}$$ (16)

which leads to

$$\begin{array}{c} \sum_{i<j}\rho_{ijk}^{2}=N. \end{array}$$ (17)

The right-hand side of Eq (15) is therefore the same for every *k*, so its left-hand side, which is in fact the inner product of two vectors in $\left(\begin{array}{c} n\\ 2 \end{array}\right)$-dimensional real space, must also not depend on *k*. The *ij*th component of one of the two vectors involved in this inner product is $N^{-1}\rho_{ijk}^{2}$, so all *d* such vectors are coplanar, since by Eq (17) they all lie on the $\left(\begin{array}{c} n\\ 2 \end{array}\right)$-dimensional plane ∑_*i* < *j*_*x*_*ij*_ = 1. In order for the inner product to have the same value regardless of *k*, the vector of *ij*th component $r_{ij}^{-3}$ must therefore be orthogonal to this plane. That is, we must have

$$\begin{array}{c} \sum_{i<j}r_{ij}^{-3}N^{-1}\left(\rho_{ijk}^{2}-\rho_{ij\ell }^{2}\right)=0, \end{array}$$ (18)

where *k*, *ℓ* are any two of the *d* dimensions. In the formulation that follows we use *k* = 1, *ℓ* = 2.

Determining the *α*_*k*_’s directly from Eq (18) and the equality constraint is not possible, so we resort to the following nonlinear programming problem instead.

$$\begin{array}{cc} \text{minimize}\; & {\left(\sum_{i<j}r_{ij}^{-3}N^{-1}\left(\rho_{ij1}^{2}-\rho_{ij2}^{2}\right)\right)}^{2} \end{array}$$ (19)

$$\begin{array}{cc} \text{subject}\;\text{to}\; & \sum_{k}\alpha_{k}^{2}=d, \end{array}$$ (20)

$$\begin{array}{ccc} & \alpha_{k}>0. & \forall k\in \{1,\ldots ,d\} \end{array}$$ (21)

Solving this problem will return *α*_*k*_’s that approximate Eq (18) as well as possible. Even if a good approximation is returned, it must be kept in mind that only first-order necessary conditions are being taken into account. The second-order necessary and sufficient conditions, which involve the second derivatives of SC, are not. Further methodological steps must then be taken, as detailed in Experimental setup along with the necessary tool set. Henceforth, we refer to the optimization problem given in Eqs (19)–(21) simply as Problem P.

### Further remarks

Another consequence of Eq (17) is that

$$\begin{array}{c} \sum_{i<j}r_{ij}^{2}=N\sum_{k}\alpha_{k}^{2}, \end{array}$$ (22)

which allows *g* to be rewritten as

$$\begin{array}{c} g={\left(N\sum_{k}\alpha_{k}^{2}\right)}^{1/2}. \end{array}$$ (23)

## Experimental setup

Solving Problem P to discover the *α*_*k*_’s is the centerpiece of our approach. Several candidate sets of these scaling factors can be obtained by solving the problem repeatedly in a sequence of random trials, each one first selecting an initial point for the minimization and then attempting to converge to a set of *α*_*k*_’s for which the problem’s objective function is locally minimum. The resulting scaling-factor sets can then be pitched against one another, engaging the user’s knowledge of the data set for the selection of a small set of candidates (perhaps even a single one) to carry on with.

Because clustering is an approach to data analysis that depends strongly on a domain expert’s knowledge of and familiarity with the data set, uncertainties during the process of selecting appropriate scaling-factor sets from those turned up by solving Problem P are inevitable. To illustrate some strategies to deal with this, in Results and in Discussion we address our experience with analyzing five well-known benchmarks in light of SC. Dealing with these data sets has of course been greatly facilitated by the availability of the reference partition into clusters for each one. This will not be available in a real-world scenario, except perhaps in some fragmentary form, but in our discussion of the benchmarks we attempt to provide viewpoints that may be useful even then.

We continue with the presentation of the benchmarks we use, and of the tools, algorithms, and evaluation methods we enlist. All computational tasks were implemented within the Mathematica 13 system or in the R language. The essential code we used is available online as S1 File.

### Data sets

The five data sets we use are listed in Table 1, along with crucial information on them. We divide them into two groups, based on our experience in handling them, particularly on the difficulty in obtaining good partitions. The first group contains those for which it has proven possible to obtain partitions that approximate the corresponding reference partition well. The second group contains those for which approximating the reference partition, even if only reasonably, has proven harder.

**Table 1:** Data sets used and their properties.

Data set number of clusters	Number of samples (*n*_orig_)	Number of unique samples (*n*)	Number of actual/original dimensions (*d*/*d*_orig_)	Number of dimensions with missing values (*d*_miss_)
Iris, 3	150	149	4/4	0
BCW, 2	699	465	9/9	1
BC-DR3, 4	62	62	3/496	392
BNA-DR3, 2	1372	1348	3/4	0
BCW-Diag-10, 2	569	569	10/30	0

The first group has two members, the Iris data set (downloaded from [16] and then corrected to exactly match the data in the original publication [17]; sample 35 was changed from 4.9 3.1 1.5 0.1 to 4.9 3.1 1.5 0.2, sample 38 from 4.9 3.1 1.5 0.1 to 4.9 3.6 1.4 0.1) and BCW, the original version of the Wisconsin breast cancer data sets [18].

The second group comprises three data sets, viz.: BC-DR3, which comes from the version of Perou et al.’s breast cancer data set [19] compiled and made available by the proponents of scaling by the $\sigma_{k}^{\text{pool}}$’s mentioned in Introduction (see the online supplementary data for their publication [5]); BNA-DR3, from a data set containing wavelet-transform versions and the entropy of banknote images for authentication [20]; and BCW-Diag-10, from the so-called diagnostic version of the Wisconsin breast cancer data sets [21].

The three data sets in the second group have fewer dimensions than originally available, which is indicated in Table 1 by the *d* < *d*_orig_ values on the fourth column. We reduced these data sets’ numbers of dimensions as an attempt to make clustering succeed better than it would otherwise. In two cases this is indicated by the “DR3” in the data sets’ names, which refers to dimensionality reduction by adopting the first three principal components output by principal component analysis (PCA) [22] on the data after centering (but not scaling) the samples. This was done in the R language, using function prcomp with options center = T and scale = F. The resulting BC-DR3 and BNA-DR3 retain 35.85% and 97.02% of the original variance, respectively.

The third case is that of BCW-Diag-10, which contains only the first 10 of the original 30 dimensions. Each sample in this data set is an image and each dimension is a statistic computed on that image. The 10 dimensions we use are mean values.

As per the second and third columns in the table, three of the data sets (Iris, BCW, and BNA-DR3) contain more samples (*n*_orig_) than unique samples (*n*). The difference corresponds to duplicates, which were discarded so that SC, and consequently Problem P, could be defined properly. The table’s fifth column is also worthy of attention, since it gives for each data set the number of dimensions (*d*_miss_) for which missing values are to be found. Such values were synthesized in the Wolfram Mathematica 13 system, using function SynthesizeMissingValues with default settings. This caused no further duplicates to appear and, in the case of BC-DR3, was of course done before PCA.

### Computational tools and algorithms

For each of the data sets in Table 1, first we ran 1000 trials, each one aiming to obtain a scaling-factor candidate set by solving Problem P. We did the required optimization in the Wolfram Mathematica 13 system, using function FindMinimum, mostly with default settings, to find a local minimum of the problem’s objective function for each trial. Our only choices of a non-default setting for FindMinimum were the following: for each trial we specified an initial point in [0.5, 1.5]^*d*^, selected uniformly at random by an application of function RandomReal to each dimension; we allowed for a larger number of iterations with the MaxIterations -> 5000 option; and we precluded any symbolic manipulation with Gradient -> “FiniteDifference” (because Problem P is strongly data-dependent, allowing Mathematica to perform the symbolic manipulations that come so naturally to it can quickly lead to memory overflow).

On occasion we have noticed that coding the constraint in Eq (21) as is can lead to division-by-zero errors. We thus avoided this by expressing the constraint as *α*_*k*_ ≥ 10^−5^ instead of *α*_*k*_ > 0. Still regarding errors during minimization, FindMinimum can also fail by not attaining convergence within the specified maximum number of iterations. This error can take more than one form, but in general we have observed it in no more than 0.2% of the trials for each data set. When a failure of this type does occur a solution is still output, but in our experiments such outputs were discarded when compiling results.

One crucial step in this study is of course partitioning the data into clusters after they have been appropriately scaled. We performed this step in the R language, using in all cases the k-means method as implemented in function kmeans. Because k-means has certain randomized components, we first set a fixed seed, via set.seed(1234), to facilitate consistency checks. Function kmeans receives as input the scaled version of the *n*_orig_ × *d* data matrix *X* and also the desired number of clusters (the same as in the data set’s reference partition). For the outcomes we report in Results, scaling happened according to one of four possibilities: either each *X*_*ik*_ remained unchanged (no scaling), or it became one of (1/*σ*_*k*_)*X*_*ik*_, $\left(1/\sigma_{k}^{\text{pool}}\right)X_{ik}$, or (*α*_*k*_/*σ*_*k*_)*X*_*ik*_. The latter is scaling as indicated by some random trial with Problem P.

### Partition evaluation

A great variety of criteria and measures exist to evaluate the partition that results from clustering. A comprehensive review of the state of the art as of 2005 is available [23] and includes the well-known Adjusted Rand Index (ARI) [24]. The current partition evaluation landscape includes new ARI variants [25] and also important novel additions, of which we single out Adjusted Mutual Information (AMI) [26], itself a class of variants of the same underlying idea. All ARI and AMI variants are similar to one another in more than one respect. For example, they all provide corrections for “chance” relative to their original versions, viz., the Rand Index (RI) [27] and Mutual Information (MI) [28]. Moreover, at bottom they all rely on counting the number of samples that concomitantly belong to each possible pair of clusters, one from the reference partition, the other from the obtained partition. However, beyond these superficial similarities ARI variants are deeply different from AMI variants: ARI variants use those sample counts to categorize sample pairs on the basis of how they stand relative to the two partitions, while AMI variants use them to acquire an information-theoretic take on how the two partitions relate to each other. In this study we use one of the recent ARI variants [25] and one of the AMI variants [26].

The ARI variant we use is the one that seems most appropriate in the present context, which is that every possible resulting partition of a data set by the clustering algorithm in use must have a fixed number of clusters (“fnc,” used in notations henceforth). That this is clearly the case follows from our use of k-means described above. The ARI variant is

$$\begin{array}{c} {\text{ARI}}_{\text{fnc}}=\frac{\text{RI}-E_{\text{fnc}}\left[\text{RI}\right]}{1-E_{\text{fnc}}\left[\text{RI}\right]}, \end{array}$$ (24)

where E_fnc_[RI] is the expected value of RI given the fixed number of clusters condition.

Letting *C* denote the number of clusters that any obtained partition will have, the formulas for RI and E_fnc_[RI] are

$$\begin{array}{c} \text{RI}={\left(\begin{array}{c} n_{\text{orig}}\\ 2 \end{array}\right)}^{-1}\left(\text{TS}+\text{TD}\right) \end{array}$$ (25)

and

$$\begin{array}{c} E_{\text{fnc}}\left[\text{RI}\right]=UV+\left(1-U\right)\left(1-V\right), \end{array}$$ (26)

with

$$\begin{array}{c} U={\left\{\begin{array}{c} n_{\text{orig}}\\ C \end{array}\right\}}^{-1}\{\begin{array}{c} n_{\text{orig}}-1\\ C \end{array}\} \end{array}$$ (27)

and

$$\begin{array}{c} V={\left(\begin{array}{c} n_{\text{orig}}\\ 2 \end{array}\right)}^{-1}\left(\text{TS}+\text{FD}\right). \end{array}$$ (28)

In Eqs (25) and (28), TS (for “true similar”) counts the number of sample pairs that are in the same cluster according to the obtained partition and in the same cluster according to the reference partition; TD (“true dissimilar”) counts pairs that are split between different clusters according to both the obtained partition and the reference partition; and FD (“false dissimilar”) counts those that are split between different clusters according to the obtained partition but are in the same cluster according to the reference partition. Curly brackets are used in Eq (27) to denote Stirling numbers of the second kind. ARI_fnc_ equals at most 1, which happens for $\text{TS}+\text{TD}=\left(\begin{array}{c} n_{\text{orig}}\\ 2 \end{array}\right)$ (i.e., when the reference partition and the obtained partition are identical).

As for the AMI variant we use, first denote the reference partition by R and the obtained partition by O. All AMI variants are based on the joint probability *p*_*RO*_ that a randomly chosen sample is found in reference cluster R∈R and in obtained cluster O∈O, given by *p*_*RO*_ = |*R* ∩ *O*|/*n*_orig_, and on its marginals, given by *p*_*R*_ = |*R*|/*n*_orig_ and *p*_*O*_ = |*O*|/*n*_orig_. These probabilities lead to one joint Shannon entropy and two marginals,

$$\begin{array}{c} H\left(R,O\right)=-\sum_{R\in R}\sum_{O\in O}p_{RO}\;\text{log}\;p_{RO}, \end{array}$$ (29)

$$\begin{array}{c} H\left(R\right)=-\sum_{R\in R}p_{R}\;\text{log}\;p_{R}, \end{array}$$ (30)

$$\begin{array}{c} H\left(O\right)=-\sum_{O\in O}p_{O}\;\text{log}\;p_{O}, \end{array}$$ (31)

and from these to the definition of MI,

$$\begin{array}{c} \text{MI}=H(R)+H(O)-H(R,O). \end{array}$$ (32)

The correction for chance leading from MI to any of the AMI variants follows the same pattern as Eq (24), where the 1 in the denominator is an upper bound on RI. Thus, recognizing that an upper bound on MI is max{H(R),H(O)}, the AMI variant we use, now denoted by AMI_max_, is given by

$$\begin{array}{c} {\text{AMI}}_{\text{max}}=\frac{\text{MI}-E[\text{MI}]}{\text{max}\{H(R),H(O)\}-E[\text{MI}]}. \end{array}$$ (33)

In this expression, E[MI] is the expected value of MI under the same assumption used in [24] for the original definition of the ARI [26]. Assuming the randomness model underlying the definition of ARI_fnc_ would be preferable, but that generalization seems as yet unavailable. AMI_max_ equals at most 1, which happens for H(R)=H(O)=H(R,O), that is, for *p*_*RO*_ = 0 whenever *R* ≠ *O* (i.e., when R and O are identical).

## Results

All our results are in reference to the data sets listed in Table 1 and are summarized in Table 2. In this table, the values of ARI_fnc_ and AMI_max_ resulting from the use of k-means are given for each of several scaled versions of the *n*_orig_ × *d* data matrix *X* that corresponds to each data set. There are four schemes in each case: the no-scaling scheme, in which each *X*_*ik*_ is used directly as it appears in the data matrix; the scheme that makes use of the standard deviation in each dimension, in which *X*_*ik*_ is scaled by 1/*σ*_*k*_; the scheme that uses pooled standard deviations instead, in which *X*_*ik*_ is scaled by $1/\sigma_{k}^{\text{pool}}$; and the scheme that uses scaling factors obtained by solving Problem P, in which *X*_*ik*_ is scaled by *α*_*k*_/*σ*_*k*_ for the resulting *α*_*k*_.

**Table 2:** Performance of k-means, according to ARI_fnc_ and AMI_max_, on various scaled versions of the data sets in Table 1.

Data set	No scaling	Scaling by 1/*σ*_*k*_	Scaling by $1/\sigma_{k}^{\text{pool}}$	Scaling by *α*_*k*_/*σ*_*k*_
	ARI_fnc_			
Iris	0.728	0.621	0.886	0.571–0.904
BCW	0.840	0.825	0.825	0.189–0.877
BC-DR3	0.492	0.518	0.518	(−0.021)–0.535
BNA-DR3	0.050	0.023	0.023	(−0.000)–0.659
BCW-Diag-10	0.456	0.673	0.673	0.633–0.655
	ARI_max_			
Iris	0.748	0.654	0.862	0.605–0.878
BCW	0.731	0.711	0.711	0.094–0.785
BC-DR3	0.637	0.653	0.653	0.089–0.663
BNA-DR3	0.029	0.010	0.010	(−0.000)–0.546
BCW-Diag-10	0.375	0.556	0.556	0.503–0.530

ARI_fnc_ and AMI_max_ values for the latter type of scaling are presented in Table 2 as intervals, indicating in each case the lowest and the highest value observed in the 1000 random trials with Problem P (slightly fewer trials if optimization errors happened). For BC-DR3 and BNA-DR3, the intervals for ARI_fnc_ begin at slightly negative values (indicating that RI < E_fnc_[RI]). The same holds for BNA-DR3 in regard to the intervals for AMI_max_ (indicating that MI < E[MI]).

**Fig 1:**
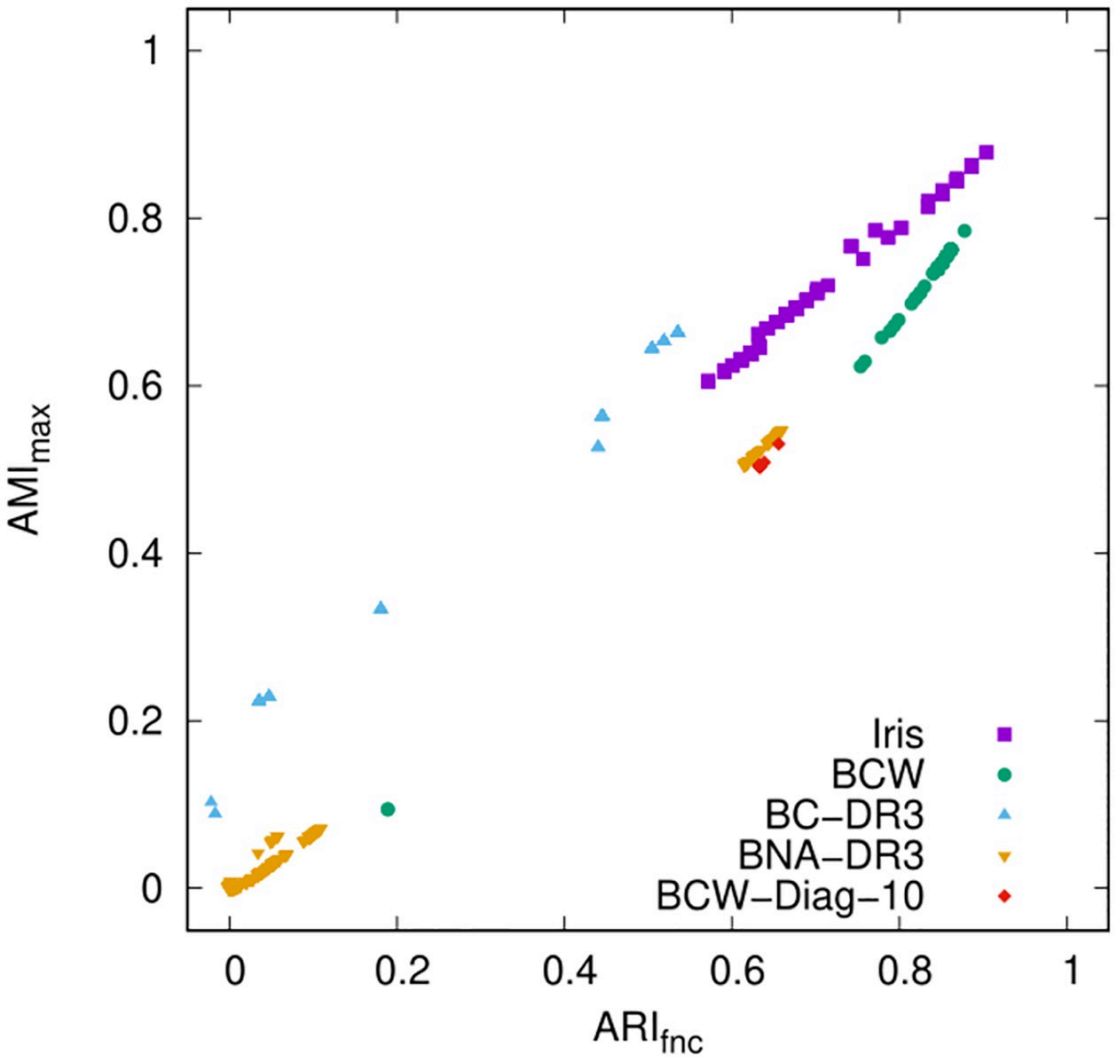
ARI_fnc_ versus AMI_max_ for all partitions resulting from scaling as in the rightmost column of Table 2.

Interestingly, despite the fundamental theoretical differences underlying the definitions of ARI_fnc_ and AMI_max_, for each data set their values in Table 2 are in a sense consistent with each other. Consider, for example, the Iris data set and the first three scaling schemes: both ARI_fnc_ and AMI_max_ have the lowest value when scaling by 1/*σ*_*k*_ is used, the next higher value under no scaling, and the highest value when scaling by $1/\sigma_{k}^{\text{pool}}$ is used. A similar type of consistency is observed for the other data sets as well. Not only this, but as we focus on the values resulting from the several instances of scaling by *α*_*k*_/*σ*_*k*_ they seem to grow approximately linearly with each other, as illustrated in the scatterplot of Fig 1. What this persistent consistency suggests is that both ARI_fnc_ and AMI_max_ are capturing the same qualities, so to speak, of the obtained partitions as they relate to the reference partitions. Therefore, henceforth we refer solely to ARI_fnc_ when discussing the results in Table 2.

**Fig 2:**
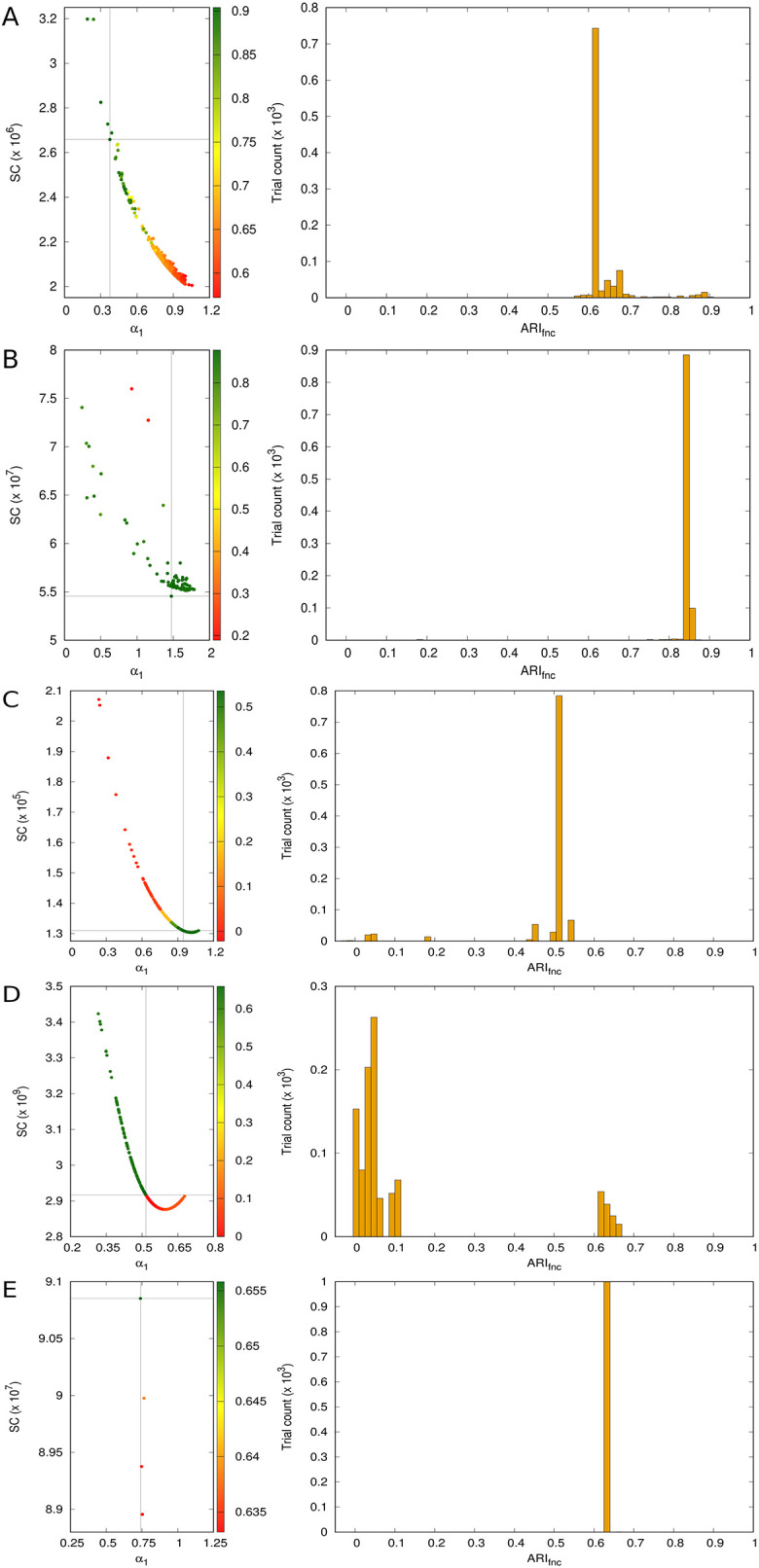
Results of the random trials with Problem P on Iris (A), BCW (B), BC-DR3 (C), BNA-DR3 (D), and BCW-Diag-10 (E), expanding on the summary given on the rightmost column of Table 2. Each point on each left panel corresponds to a trial and is color-coded according to the accompanying palette to reflect the value of ARI_fnc_ it leads to by way of clustering with k-means. The point leading to the highest ARI_fnc_ value is marked by the crosshair in the panel. Each right panel provides a view of how ARI_fnc_ is distributed over all pertaining trials.

The intervals on the rightmost column of Table 2 are supplemented by the panels in Fig 2, where each row of panels (rows A through E) corresponds to one of the data sets. Such panels allow viewing the various ARI_fnc_ values inside those intervals from different perspectives. The left panel on each row is a plot of SC against *α*_1_ for all trials on the corresponding data set. The choice of *α*_1_ is completely arbitrary and meant only to offer a glimpse into how SC depends on the *α*_*k*_’s turned up by solving Problem P. Points are color-coded to indicate how their ARI_fnc_ values relate to one another. The right panel on each row allows viewing such values as a histogram.

## Discussion

Table 2 confirms, for the selection of data sets we are considering, what by and large has been known for a long while. That is, that scaling by 1/*σ*_*k*_ can sometimes be worse than simply attempting to partition the data in *X* into clusters without any scaling. In the table, this is the case mainly of the Iris data set. Table 2 also confirms what has been known since the recent introduction of scaling by $1/\sigma_{k}^{\text{pool}}$, which is that proceeding in this way, once again in the case of Iris, leads to superior performance. The table goes farther than this, however, since it also makes clear that the fallback role of *σ*_*k*_ as a surrogate for $\sigma_{k}^{\text{pool}}$ in the approach of [5] may be taken more frequently than initially realized. This is shown in the table for all but the Iris data set.

But the most relevant contribution of the results in Table 2 is the realization that in almost all cases the best performing set of *α*_*k*_’s for each data set performs strictly better than the other three alternatives. The only exception is the BCW-Diag-10 data set, although in this case every one of the sets of *α*_*k*_’s can be said to lie, so to speak, in the same ballpark as 1/*σ*_*k*_ (or $1/\sigma_{k}^{\text{pool}}$). In fact, the plots in Fig 2(E) strongly suggest that scaling the data for BCW-Diag-10 by the outcome of virtually any of the random trials with Problem P would be equally acceptable. This would be so even if a reference partition (and hence ARI_fnc_ values) had not been available, because comparing the obtained partitions with one another would already suffice.

Of course, the latter is based almost entirely on the highly concentrated character of the ARI_fnc_ histogram in Fig 2(E), which to a degree is also true of Fig 2(B) and 2(C), which refer to the BCW and BC-DR3 data sets, respectively. For each of these two data sets, comparing the partitions resulting from the random trials with Problem P with one another, and adopting any of those that by the ARI_fnc_ histogram seem not only to be one and the same but also to recur very frequently during the trials, would lead to equally acceptable scaling decisions.

This leaves us with the Iris and BNA-DR3 data sets. In these two cases, choosing the set of *α*_*k*_’s to use out of those produced by the random trials with Problem P by simply comparing the obtained partitions and looking for a consensus with strong support would lead to disastrous results. This is clear from the ARI_fnc_ histograms in Fig 2(A) and 2(D), which peak significantly to the left of the best values attained in the trials. Beyond comparing obtained partitions, one must therefore also use one’s knowledge of the domain in question and look at what they are doing to the data. The guiding principle to be used is essentially in the spirit of our discussion in Shape complexity: in the end, the candidate set of *α*_*k*_’s to be chosen must lead to a partition that makes sense, either visually or by inspection of the “midrange” distances between samples, those that can be more easily mistaken for intracluster when they are intercluster or conversely.

We proceed with the aid of Table 3, which lists each 1/*σ*_*k*_ and each *α*_*k*_/*σ*_*k*_ (this one for the highest ARI_fnc_ listed in Table 2) for Iris and BNA-DR3. Note, for the Iris data set, that *k* = 1 and *k* = 3 are the dimensions for which switching from scaling by 1/*σ*_*k*_ to scaling by *α*_*k*_/*σ*_*k*_ provides the greatest scaling-factor reduction and amplification (given, in fact, by the value of *α*_*k*_), respectively. For the BNA-DR3 data set, dimension *k* = 1 has its weight on distances strongly reduced in moving from the former scaling scheme to the latter, while for both *k* = 2 and *k* = 3 the scaling factor is amplified by about the same proportion.

**Table 3:** Scaling factors used in Figs 3 (Iris) and 4 (BNA-DR3). The *α*_*k*_’s are the ones leading to the highest values of ARI_fnc_ in the intervals on the rightmost column of Table 2.

*k*	1/*σ*_*k*_	*α*_*k*_/*σ*_*k*_	1/*σ*_*k*_	*α*_*k*_/*σ*_*k*_
	Iris		BNA-DR3	
1	1.207	0.453	0.141	0.073
2	2.294	1.291	0.327	0.373
3	0.566	0.859	0.477	0.571
4	1.311	1.459		

**Fig 3:**
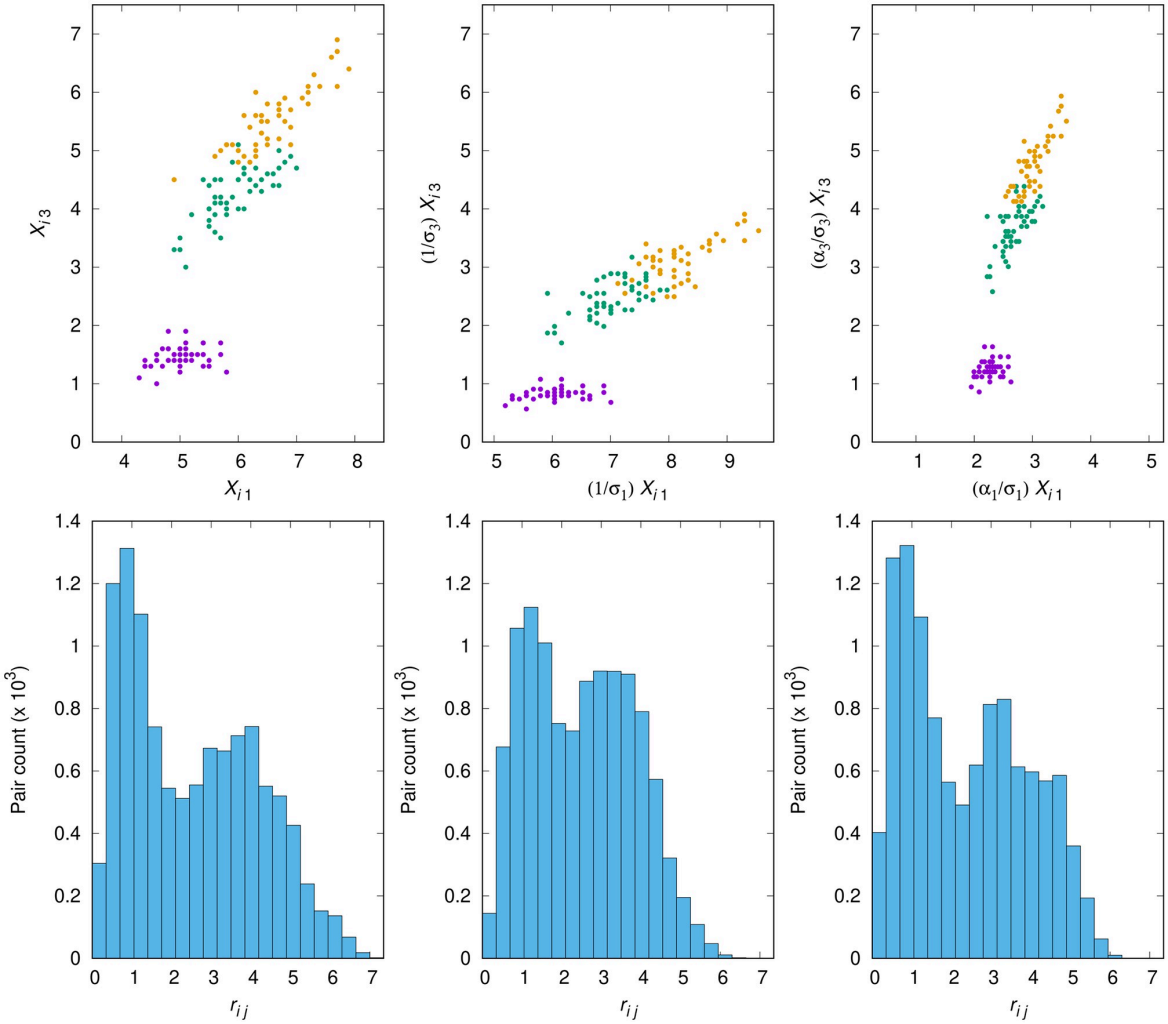
Reference partition for the Iris data set (leftmost column of panels) and the effects of two scaling schemes: Scaling by 1/*σ*_*k*_ (middle column) and scaling by *α*_*k*_/*σ*_*k*_ (rightmost column), with factors as in Table 3. Effects can be seen both with respect to the shape of the data set (top row of panels, all plots drawn to the same scale) and to the distribution of distances between samples (the *r*_*ij*_’s; bottom row, all plots drawn to the same scale).

For the Iris data set, in Fig 3 we give six panels. These are arranged in three columns, the leftmost one dedicated to the data set’s reference partition, each of the other two to a different scaling scheme (scaling by 1/*σ*_*k*_ and scaling by *α*_*k*_/*σ*_*k*_, with factors as in Table 3). The top panel in each column contains a scatterplot of the samples, each color-coded for the data set’s three classes, as represented by dimensions *k* = 1 and *k* = 3 (cf. the discussion above in reference to Table 3). The bottom panel is a histogram of the *r*_*ij*_’s, the distances between samples in *d*-dimensional real space. It is important to emphasize that, in regard to the leftmost column, and unlike what happens with the other two, the scatterplot in it is color-coded to reflect the reference partition, not the obtained partition that results from the no-scaling scheme.

As we examine the scatterplots in the figure we see that scaling by 1/*σ*_*k*_ stretches dimension *k* = 1 excessively just as dimension *k* = 3 is excessively shrunk, resulting in more confusion between the two clusters that are not linearly separable. We also see why scaling by *α*_*k*_/*σ*_*k*_ as in Table 3 is a better choice: the previous stretching of dimension *k* = 1 and the shrinking of dimension *k* = 3 are both undone, though to different degrees, which allows some of the previously added confusion to be reverted. Examining the distance histograms reveals that scaling by 1/*σ*_*k*_ causes distances to become more concentrated, lengthening some of the smallest ones and shortening some of the largest. So another way to view the further confusion added by this scaling scheme is to recognize that it affects the already potentially problematic midrange distances. Scaling by *α*_*k*_/*σ*_*k*_ restores the overall appearance of the no-scaling histogram (the one in the leftmost column), but seemingly with sharper focus around those distances. This is important because, as we know from Table 2, scaling the Iris data set by the *α*_*k*_/*σ*_*k*_ factors of Table 3 improves not only on the use of the 1/*σ*_*k*_ factors but also on the no-scaling scheme, on which k-means already performs more than reasonably well.

**Fig 4:**
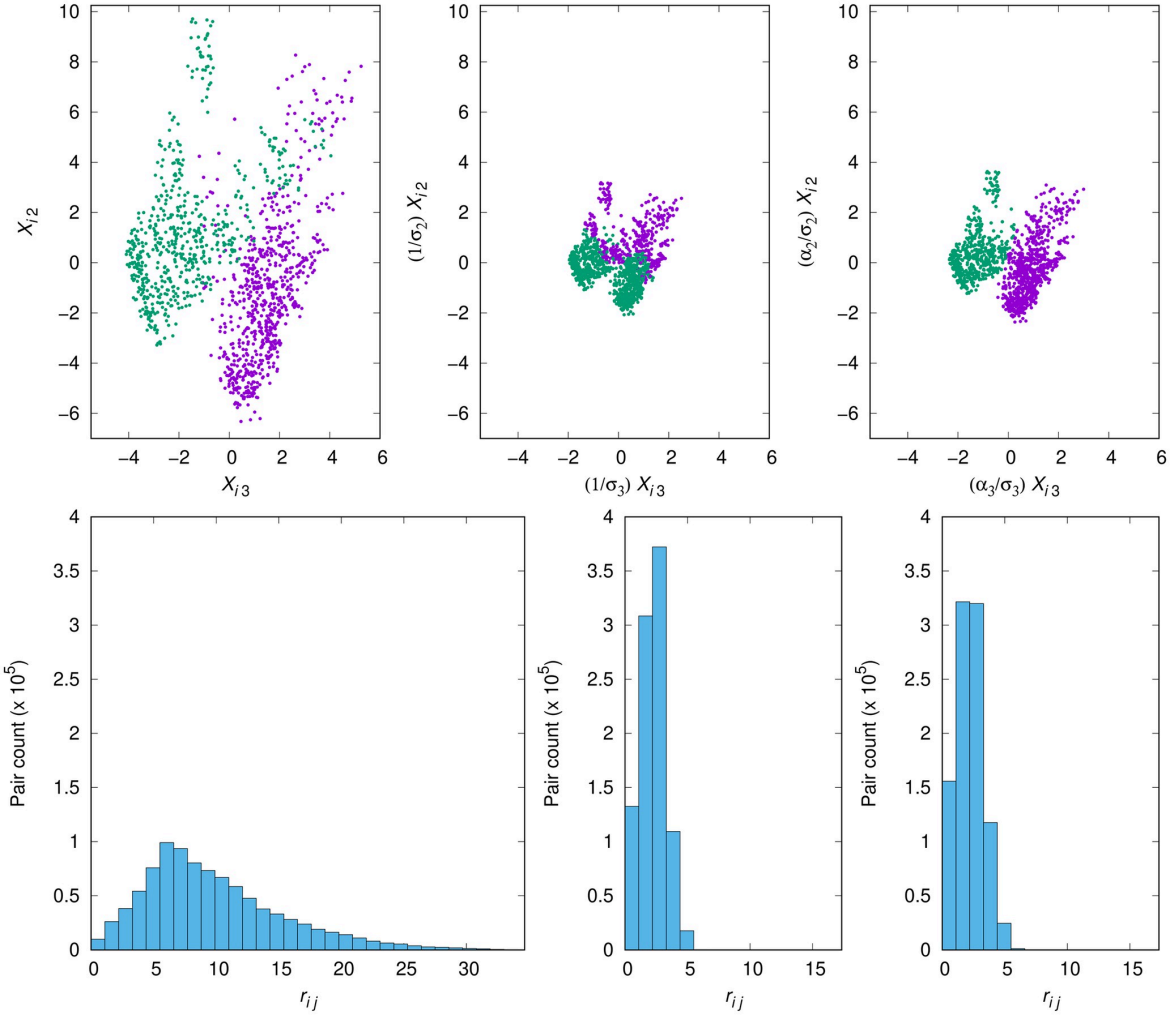
As in Fig 3, now for the BNA-DR3 data set.

Fig 4 has the same six panels as Fig 3, and also identically arranged, but now referring to the BNA-DR3 data set. This data set provides a much more striking contrast between the two scaling schemes given in Table 3 than Iris, as per Table 2 the ratio of the ARI_fnc_ value that scaling by *α*_*k*_/*σ*_*k*_ yields to that of scaling by 1/*σ*_*k*_ is about 28.65.

The most direct pictorial evidence we have of this comes from comparing the middle and rightmost scatterplots of Fig 4 with each other, having the leftmost one as the reference partition. Because the ratio of *α*_*k*_/*σ*_*k*_ to 1/*σ*_*k*_ (i.e., the value of *α*_*k*_) is slightly higher than only 1.1 for both *k* = 2 and *k* = 3 (once again, cf. our earlier comment on this), what really accounts for the very significant difference between the two scaling schemes has to do with dimension *k* = 1, for which a ratio of about 0.517 ensues. Visually inspecting the two obtained partitions vis-à-vis the reference partition provides immediate confirmation of how crucial this shrinking of dimension *k* = 1 is. As with the Iris data set, inspecting the histograms in the figure provides insight similar to the one we gleaned in that case. Even though the middle and rightmost histograms, corresponding respectively to scaling by 1/*σ*_*k*_ and *α*_*k*_/*σ*_*k*_, may seem similar to each other particularly when viewed in comparison to the no-scaling histogram (the leftmost one), closer inspection tells a different story. That is, moving from scaling by 1/*σ*_*k*_ to scaling by *α*_*k*_/*σ*_*k*_ seems to restore some of the no-scaling histogram’s slow descent from its peak through the midrange distances. This comes about by virtue of both a lower peak and the appearance of some residual pair count beyond the *r*_*ij*_ = 5 bar when the additional *α*_*k*_ factor is put to use. This is curious, especially as we note from Table 2 that no scaling and scaling by 1/*σ*_*k*_ both lead k-means to essentially the same poor performance. This seems to be suggesting that, in the no-scaling scheme, such poor performance is to be attributed essentially to the excessive spread of distances.

## Conclusions

In this paper we have revisited the problem of scaling a data set’s dimensions to facilitate clustering by those methods that, like k-means, make explicit use of distances between samples. For each dimension *k*, we have framed our study as the determination of a scaling factor *α*_*k*_ > 0 to be applied on top of the customary division by *σ*_*k*_, the standard deviation of the data in that dimension. That is, we have targeted a scaling factor of the form *α*_*k*_/*σ*_*k*_. Our guiding principle has been to focus on the effects of scaling the data on the multidimensional shapes that ensue: essentially, we have equated any facilitation of the clustering task with mistaking intercluster distances for intracluster distances (or conversely) as seldom as possible. Because we normally think of the former type of distances as being large, and the latter as being small, we have aimed our efforts at midrange distances.

To make such notions precise, we enlisted the shape complexity of the scaled data, given by SC, which depends heavily on the data matrix *X* (as a constant) and on the various *α*_*k*_’s (as variables). The function SC embodies a lot of the tension between large and small distances between samples and, as such, allows midrange distances to be characterized as being the equilibrium between extremes that occurs at those *α*_*k*_’s for which the gradient of SC is zero. We have viewed such scaling-factor sets as candidates, each one obtained by solving Problem P, given in Eqs (19)–(21), from a randomly chosen initial point.

Our results can be summarized very simply: for all data sets we tackled, generating scaling-factor candidate sets via Problem P has yielded at least one set for which scaling by *α*_*k*_/*σ*_*k*_ (as opposed to 1/*σ*_*k*_) leads to strictly better performance (with one single exception, where “strictly better” becomes “comparable”). The overall method cannot be used as a blind procedure, though, since in at least two cases we came across the need for carefully considered visual inspections of the scaled data, perhaps even of their distance histograms. In fact, this exploration of possibilities is in essence what cluster analysis is about. The role of Problem P is to provide well-founded sets of scaling factors to experiment with.

In spite of the radial invariance of SC, which we used to constrain the *α*_*k*_’s when formulating Problem P, the number of possibilities outside the reach of Problem P is limitless. Suppose, for example, that we use the following alternative formulation of the nonlinear programming problem.

$$\begin{array}{c} \text{maximize}\;\text{SC} \end{array}$$ (34)

$$\begin{array}{cc} \text{subject}\;\text{to}\;\alpha_{k}\geq 10^{-5}. & \forall k\in \{1,\ldots ,d\} \end{array}$$ (35)

In significant ways this is still in the spirit of Problem P, even though it limits the notion of a gradient-zero point to those that correspond to local maxima of SC. We mention this particular formulation because solving it for the Iris data set, as explained in Experimental setup for Problem P (now with FindMaximum substituting for FindMinimum and selecting the initial points from [10^−5^, 1]^*d*^), yielded ARI_fnc_ = 0.922 in the best case, with *α*_1_ = *α*_2_ = 10^−5^, *α*_3_ = 5.09372 × 10^17^, and *α*_4_ = 2.48504×10^17^. This is an interesting outcome, and not only because it surpasses the best result reported in Table 2 (ARI_fnc_ = 0.904). What these *α*_*k*_’s are saying is: reduce the importance of dimensions *k* = 1 and *k* = 2 as far as allowed by the constraint in Eq (35) while dimensions *k* = 3 and *k* = 4 are very strongly amplified. This is to a degree already what Table 3 is suggesting, though only in relation to scaling by 1/*σ*_*k*_ and moreover much more timidly. Alternative formulations like this, and the surprising results they may lead to, serve to illustrate the rich store of possibilities for shape complexity-based cluster analysis. Additional investigations to further explore SC and its role in helping determine appropriate scaling factors for any given data set could well be worth the effort.

Such tantalizing possibilities notwithstanding, it is critical that Problem P be solvable without too much computational effort. All our results refer to data sets that are essentially manageable when considering both their numbers of samples and numbers of dimensions. The performance of k-means clustering on them, as measured by ARI_fnc_, ranges from very poor to well above average, so calling them “manageable” refers not at all to how amenable to clustering by k-means they are, but rather to the possibility of solving Problem P for them multiple times within reasonable bounds on the computational resources required. In this regard, we note that, already for the precursor of the BC-DR3 data set, with *n*_orig_ = 62 and *d*_orig_ = 496, solving one trial with Problem P is expected to take a few hundred hours. While in this case we resorted to the BC-DR3 version for different reasons (distances become ever less meaningful as the number of dimensions grows and clustering algorithms perform ever more poorly), the issue remains that a data set with substantially more samples than the ones we have considered is expected to be burdensome to solve.

There are options to be considered, though. Modern solvers of linear as well as nonlinear optimization problems already incorporate many functionalities for automatically going into parallel-processing mode whenever possible. This is true also of the Mathematica 13 system we used and is bound to provide more speedup as more processor cores are brought in. Beyond this, open-source frameworks are beginning to become available (e.g., [29]) and are poised to make a difference by targeting scalable parallel performance as a main goal. Indispensable though these technological possibilities and improvements will be, a crucial ingredient will almost certainly be the use of techniques to not only reduce the number of dimensions in the data, but also the number of samples. The latter has been studied for a while now (e.g., [30]), often in the wake of insufficient parallel infrastructure, but in view of Problem P acquires special significance even as we consider the great proliferation of parallel hardware as well as software since then.

## Supporting information

S1 File (GZ)
