# Supplementary material for: Shape complexity in cluster analysis
Source: PLoS One. 2023 May 26;18(5):e0286312. doi: 10.1371/journal.pone.0286312 (PMC10218739; doi:10.1371/journal.pone.0286312)
Supplement: S1 File — (GZ) [file pone.0286312.s001.gz › scompl-suppinfo/scompl-code.pdf]

Shape Complexity in Cluster Analysis (Essential code)

Eduardo J. Aguilar
Instituto de Ciência e Tecnologia
Universidade Federal de Alfenas
Rod. José Aurélio Vilela, 11999
37715-400 Poços de Caldas - MG, Brazil

Valmir C. Barbosa*
Programa de Engenharia de Sistemas e Computação, COPPE
Universidade Federal do Rio de Janeiro
Centro de Tecnologia, Sala H-319
21941-914 Rio de Janeiro - RJ, Brazil

Outline

This file contains nearly all the essential code (in Mathematica or in R) underlying the computational tasks in the study, along with detailed comments. It also sets the convention for naming the accompanying data files.

Some external code was also used, specifically to compute the σ_k^{pool}'s. For this the reader is referred to the supplementary data made available with the original publication [1], which include the relevant script in R. For each data set used in the study, a file `dataset-noMV-psd` containing the resulting σ_k^{pool}'s is included in the present compilation of supporting information.

*Corresponding author (valmir@cos.ufrj.br).

Essential Mathematica code

In this section we deal mostly with solving Problem P and computing the value of $\mathrm{ARI}_{\mathrm{fnc}}$ for a given obtained partition.

Problem P refers to a data set with n d-dimensional samples, assumed to be stored in file `dataset-noMVnoDups`, one sample per row, each row containing d numerical values separated by spaces. This file does not necessarily contain the original data in full, since those may have missing values (if $d_{\mathrm{miss}} > 0$, as in BCW and BC-DR3 in the study) or duplicated samples (if $n < n_{\mathrm{orig}}$, as in Iris, BCW, and BNA-DR3).

In the case of missing values, the original data are assumed to be stored in file `dataset-orig`, with the missing values temporarily filled in by the string `Missing[]`. If no values are missing, then the original data are assumed to be in `dataset-noMV`. The following commands are used to replace all occurrences of `Missing[]` by synthesized values generated by `SynthesizeMissingValues`, a function that by default models the probability density underlying the data in `dataset-orig` as a multivariate normal and samples from it during synthesis. The resulting data set is output to file `dataset-noMV`.

```
pts = Import["dataset-orig", "Table"]/. "Missing[]" -> Missing[];
pts = SynthesizeMissingValues[pts];
Export["dataset-noMV", pts, "Table", "FieldSeparators" -> " "];
```

If `dataset-noMV` contains duplicates, then these are removed and the results are saved to `dataset-noMVnoDups`. If not, then `dataset-noMV` is simply copied to `dataset-noMVnoDups`.

A first preparatory step before solving Problem P consists of computing σ_k for $1 \leq k \leq d$ from the data in `dataset-noMV` (i.e., possible duplicates do contribute to the standard deviations) and scaling the data in `dataset-noMVnoDups` by dividing them by the σ_k's as appropriate. The σ_k's are written to file `dataset-noMV-std` for further use.

```
pts = Import["dataset-noMV", "Table"];
std = StandardDeviation[pts];
Export["dataset-noMV-std", {N[std]}, "Table",
                                    "FieldSeparators" -> " "]
pts = Import["dataset-noMVnoDups", "Table"];
n = Length[pts];
d = Length[pts[[1, All]]];
For[k = 1, k <= d, k++,
    pts[[All, k]] = pts[[All, k]]/std[[k]]];
```

Further preparation follows, as given next, with the creation of tables to store precomputed values that do not depend on the α_k's (e.g., the $\binom{n}{2} \times d$ table `rho2n` for $N^{-1}\rho^2_{ijk}$) and with the definition of functions of the α_k's (e.g., `dtpr[a_]` for $\sum_{i<j} r_{ij}^{-3} N^{-1}(\rho^2_{ij1} - \rho^2_{ij2})$ and `r2[a_]` for $\sum_k \alpha_k^2$).

```
np = Binomial[n, 2];
thei = Table[0, np];
thej = Table[0, np];
c = 0;
For[i = 1, i < n, i++,
    For[j = i + 1, j <= n, j++,
        c = c + 1;
        thei[[c]] = i;
        thej[[c]] = j]];
rho = Table[pts[[thei[[c]], k]] - pts[[thej[[c]], k]],
            {c, 1, np}, {k, 1, d}];
rho2 = Table[Power[rho[[c, k]], 2],
             {c, 1, np}, {k, 1, d}];
srho2 = Table[Sum[rho2[[c, k]],
                  {c, 1, np}],
              {k, 1, d}];
rho2n = Table[rho2[[c, k]]/srho2[[k]],
              {c, 1, np}, {k, 1, d}];
dist[a_, c_] := Sqrt[Sum[Power[a[[k]]*rho[[c, k]], 2],
                         {k, 1, d}]];
distm3[a_, c_] := Power[dist[a, c], -3];
dtpr[a_] := Sum[distm3[a, c]*(rho2n[[c, 1]] - rho2n[[c, 2]]),
                {c, 1, np}];
s2[a_] := Sum[Power[dist[a, c], 2],
              {c, 1, np}];
sm1[a_] := Sum[Power[dist[a, c], -1],
               {c, 1, np}];
sc[a_] := Power[s2[a], 1/2]*sm1[a];
r2[a_] := Sum[Power[a[[k]], 2],
              {k, 1, d}];
```

Problem P is solved for each of `nInitPoints` random trials, using for each one a randomly generated d-dimensional starting point. The core of each trial is function `FindMinimum`, which uses the interior point method for constrained local minimization. Each starting point is determined with function `RandomReal` by randomly selecting a number between `aMinS` and `aMaxS` for each dimension. All `nInitPoints` points are determined in the beginning and stored in a table for further use. The code for these preparations is given next, along with the definition of the variables to be used for the α_k's (`a[1]` through `a[d]`), the value `aMin` to serve as lower bound for each of them, the maximum number of iterations (`nIter`) for each call to `FindMinimum`, and the file (`dataset-output`) to which all results are to be written.

```
nInitPoints = 1000;
aMinS = 0.5;
aMaxS = 1.5;
```

```
itpt = Table[0, nInitPoints, d];
For[p = 1, p <= nInitPoints, p++,
    For[k = 1, k <= d, k++,
        itpt[[p, k]] = RandomReal[{aMinS, aMaxS}]]];
vars = Array[a, d];
aMin = 0.00001;
nIter = 5000;
outFile = "dataset-output";
```

Code for the loop through the random trials is given next. The call to `FindMinimum` specifies the objective function of Problem P, its constraints, the starting point to be used, and the maximum number of iterations for convergence to be achieved. This call also expressly specifies, by selecting `"FiniteDifference"` as the method for gradient evaluation, that symbolic computations must not be attempted. The call to `FindMinimum` is wrapped in a call to function `Check`, which upon the return of a failure-to-converge message substitutes `failed` for the possibly unreliable results returned by `FindMinimum`.

```
For[p = 1, p <= nInitPoints, p++,
    results =
        Check[FindMinimum[{Power[dtpr[vars], 2],
                           And @@ Thread[vars >= aMin],
                           r2[vars] == d},
                          MapThread[Thread@{#1, #2} &]@
                                          {vars, itpt[[p, All]]},
                          MaxIterations -> nIter,
                          Gradient -> "FiniteDifference"],
              failed];
    If[results == failed,
       PutAppend[{results}, File[outFile]],
       Null,
       PutAppend[Join[{results[[1]]},
                      {sc[Values[results[[2, All]]]]},
                      {Values[results[[2, All]]]},
                      {itpt[[p, All]]}],
                 File[outFile]]]];
```

All results are appended to `dataset-output`, consisting of either `{failed}`, in the case of a failure, or a function of the results returned by `FindMinimum`. The latter depends on what is desired as output. In the above code, output includes the value of the objective function at the α_k's to which the minimization converged and the corresponding value of SC, as well as two further d-tuples, one with the resulting α_k's themselves, another with the starting point that was used. An example for $d = 4$ is given next.

```
{5.881973990921647*^-17, 2.104089347929662*^6,
   {0.8314519451098048, 0.9912861849495028,
    1.0517836244847891, 1.1044412936784758},
   {0.7962564860091723, 1.1754774856704548,
    1.1015427636845871, 1.1805833336760347}}
```

A simpler output, containing only the space-separated α_k's obtained for each trial that did not fail to converge, has been generated for each data set used in the study. It is available as the accompanying file `dataset-alphas`.

The final Mathematica code is for computing ARI_{fnc} for a data set's reference partition and some partition obtained after clustering, assumed to be available in files `dataset-refPart` and `dataset-obtPart`, respectively. Each of these is a single-column file containing the cluster numbers of the corresponding samples in file `dataset-noMV`. Cluster numbers start from 1. Some preparatory code is given first to import the two partitions and determine the number `nClRef` of clusters in the reference partition.

```
refPart = Import["dataset-refPart", "Table"];
nClRef = Max[refPart];
nClObt = nClRef;
obtPart = Import["dataset-obtPart", "Table"];
```

Next is the code to compute ARI_{fnc}, which is a little more general than what was needed in the study, since it allows for the number of clusters in the obtained partition (`nClObt`) to be different from `nClRef`.

```
mtrx = Table[0, nClObt, nClRef];
For[i = 1, i <= n, i++,
    p = obtPart[[i]];
    q = refPart[[i]];
    mtrx[[p, q]] = mtrx[[p, q]] + 1];
ts = Sum[Binomial[mtrx[[p, q]], 2],
         {p, 1, nClObt}, {q, 1, nClRef}];
fs = Sum[Binomial[Total[mtrx[[p, All]]], 2],
         {p, 1, nClObt}] -
     ts;
fd = Sum[Binomial[Total[mtrx[[All, q]]], 2],
         {q, 1, nClRef}] -
     ts;
td = Binomial[n, 2] -
     Sum[Binomial[Total[mtrx[[p, All]]], 2],
         {p, 1, nClObt}] -
     Sum[Binomial[Total[mtrx[[All, q]]], 2],
         {q, 1, nClRef}] +
     ts;
t = ts + fs + fd + td;
```

```
ri = (ts + td)/t;
u = StirlingS2[n - 1, nClObt]/StirlingS2[n, nClObt];
v = (ts + fd)/t;
e = u*v + (1 - u)*(1 - v);
ari = (ri - e)/(1 - e);
Join[ari, {ts, fs, fd, td}]];
```

An example of what is output in the end is given below for $n_{\text{orig}} = 150$ with nClRef $= 3$. It consists of the value of ARI_{fnc}, followed by the values of TS, FS, FD, and TD. (Note that FS is not strictly needed, since it is determined by the other three and the number $\binom{n_{\text{orig}}}{2}$ of sample pairs, but is here provided for the sake of completeness. FS is the number of "false similar" pairs, i.e., those that are in the same cluster according to the obtained partition but are split between different clusters according to the reference partition.)

```
{0.728485, 3075, 744, 600, 6756}
```

Essential R code

This section completes the essential code used in the study. It deals with running PCA when needed, applying the k-means method to partition a data set into clusters, and also with computing the value of AMI_{max} given two partitions of the data.

In cases such as those of data sets BC-DR3 and BNA-DR3 in the study, an intermediate step is needed between synthesizing missing values and removing duplicates (that is, a step before file `dataset-noMVnoDups` is obtained from `dataset-noMV`). This additional step consists in transforming the data in `dataset-noMV` by applying PCA to them, so that some of their principal components can replace the original dimensions and thereby reduce dimensionality. This is achieved by first moving `dataset-noMV` to `dataset-noMV-prePCA` and then using function `prcomp` on this file and outputting the resulting principal components to file `dataset-DR`, as shown next. This file contains $\min\{n_{\text{orig}}, d_{\text{orig}}\}$ columns, since only n_{orig} principal components exist when $n_{\text{orig}} < d_{\text{orig}}$, as in data set BC-DR3. For information on how much variance is explained by each principal component, the percentage corresponding to each of them is output to file `dataset-var`. The first d columns of `dataset-DR` are saved to the new `dataset-noMV`, which then allows `dataset-noMVnoDups` to be created. Note that $d = 3$ for both BC-DR3 and BNA-DR3.

```
pts = read.table("dataset-noMV-prePCA")
pca = prcomp(pts, center = T, scale = F)
write.table(pca$x, "dataset-DR", row.names = F, col.names = F)
eig = (pca$sdev)^2
var = eig*100/sum(eig)
write.table(t(var), "dataset-var", row.names = F, col.names = F)
```

Another essential computational task is applying the k-means method to each of the thousands of variants of the five data sets used in the study. This is achieved with function `kmeans`, to which both the data set to be clustered (assumed to be in file `dataset-scaled`, itself obtained from file `dataset-noMV` as needed) and its reference number of clusters (`nClRef`) are passed as parameters. The resulting partition is written to file `dataset-obtPart`, which is a single-column file containing the cluster numbers of the corresponding samples in `dataset-noMV`.

```
set.seed(1234)
pts = read.table("dataset-scaled")
pts.clustered = kmeans(pts, nClRef)
write.table(pts.clustered$cluster, "dataset-obtPart",
            row.names = F, col.names = F)
```

The final task is to compute the value of $\mathrm{AMI}_{\mathrm{max}}$ for a data set's reference partition and the partition resulting from clustering the data. As before, these are assumed to be available in files `dataset-refPart` and `dataset-obtPart`, respectively.

```
library("aricode")
refPart = unlist(read.table("dataset-refPart"))
obtPart = unlist(read.table("dataset-obtPart"))
ami = AMI(refPart, obtPart)
```

For the example given above with $n_{\mathrm{orig}} = 150$ in reference to $\mathrm{ARI}_{\mathrm{fnc}}$, now the output is `ami` $= 0.7483724$.

References

[1] J. Raymaekers and R. H. Zamar. Pooled variable scaling for cluster analysis. *Bioinformatics*, 36:3849–3855, 2020.
